# Inhibition of eIF2α dephosphorylation inhibits ErbB2-induced deregulation of mammary acinar morphogenesis

**DOI:** 10.1186/1471-2121-10-64

**Published:** 2009-09-15

**Authors:** Sharon J Sequeira, Huei Chi Wen, Alvaro Avivar-Valderas, Eduardo F Farias, Julio A Aguirre-Ghiso

**Affiliations:** 1 Department of Medicine and Department of Otolaryngology, Division of Hematology and Oncology, Tisch Cancer Institute at Mount Sinai, Mount Sinai School of Medicine, One Gustave L. Levy Place, New York, NY 10029, USA; 2 Department of Biomedical Sciences, School of Public Health and Gen*NY*sis Center for Excellence in Cancer Genomics, University at Albany, State University of New York, One Discovery Drive, Rensselaer, NY 12144-2346, USA; 3 Department of Biological Sciences, Life Sciences Research Building 1113, University at Albany, State University of New York, 1400 Washington Ave, Albany, NY 12222, USA

## Abstract

**Background:**

The ErbB2/Her2/Neu receptor tyrosine kinase is amplified in ~30% of human breast cancers. Phosphorylation of the translation initiation factor, eIF2α inhibits global protein synthesis and activates a stress signaling and growth suppressive program. We have shown that forced phosphorylation of eIF2α can suppress head and neck, colorectal carcinoma and multiple myeloma tumor growth and/or survival. Here we explore whether ErbB2 modulates eIF2α phosphorylation and whether forced phosphorylation of the latter can antagonize ErbB2 deregulation of mammary acinar morphogenesis.

**Results:**

We tested whether ErbB2 signaling influenced eIF2α signaling and whether enhanced phosphorylation of the latter affected ErbB2-deregulated mammary acinar development. We obtained stable MCF10A cells overexpressing wild-type (Wt) Neu/ErbB2 or a constitutively active (CA) variant via retroviral delivery or mammary tumor cells from MMTV-Neu tumors. Western blotting, RT-PCR and confocal microscopy were used to analyze the effects of ErbB2 activation on eIF2α signaling and the effect of the GADD34-PP1C inhibitor salubrinal. Wt- and MMTV-Neu cells formed aberrant acini structures resembling DCIS, while CA-ErbB2 overexpression induced invasive lesions. In these structures we found that CA-ErbB2 but not the Wt variant significantly down-regulated the pro-apoptotic gene CHOP. This occurred without apparent modulation of basal phosphorylation of PERK and eIF2α or induction of its downstream target ATF4. However, inhibition of eIF2α dephosphorylation with salubrinal was sufficient to inhibit Wt- and CA-ErbB2- as well as MMTV-Neu-induced deregulation of acinar growth. This was linked to enhanced CHOP expression, inhibition of proliferation, induction of apoptosis and luminal clearing in Wt-ErbB2 and to inhibition of cyclin D1 levels and subsequent proliferation in CA-ErbB2 cells.

**Conclusion:**

Depending on the strength of ErbB2 signaling there is a differential regulation of CHOP and eIF2α phosphorylation. ErbB2 uncouples in basal conditions eIF2α phosphorylation from CHOP induction. However, this signal was restored by salubrinal treatment in Wt-ErbB2 expressing MCF10A cells as these DCIS-like structures underwent luminal clearing. In CA-ErbB2 structures apoptosis is not induced by salubrinal and instead a state of quiescence with reduced proliferation was achieved. Treatments that stabilize P-eIF2α levels may be effective in treating ErbB2 positive cancers without severely disrupting normal tissue function and structure.

## Background

The receptor tyrosine kinase ErbB2 is over-expressed in approximately 30% of human breast carcinomas [1-3] and is a marker of poor prognosis [4]. Several transgenic mouse models over-expressing human *Her2 *or rat *Neu*, have highlighted the similarities between ErbB2/Neu-mediated oncogenesis in murine models and human breast cancer [5]. The effects of ErbB2 mediated oncogenic transformation on breast epithelial biology have been faithfully modeled using 3D reconstituted basement membrane (Matrigel) culture systems [6,7]. Activation of ErbB2 via receptor dimerization, generates tyrosine-phosphorylated recognition motifs, binding of SH2-domain signaling proteins, that transmit proliferative/survival signals via multiple pathways, including the Shc- and/or Grb2-activated Ras-Raf-MAPK and phosphatidylinositol-3-kinase (PI-3 K) pathways (reviewed in [8]). Although the mechanisms by which ErbB2 deregulates these pro-mitogenic/survival signaling networks have been well characterized, how ErbB2 signaling might influence the cellular translation machinery to propagate its oncogenic effects is still poorly understood.

The initiation factor eIF2 participates in a rate-limiting step during the initiation of translation, namely the formation of the ternary complex (TC) and AUG initiation codon recognition. The role of eIF2α phosphorylation during transformation by oncogenes like ErbB2 is poorly understood. GTP-bound eIF2 interacts with the initiator methionyl tRNA to form a ternary complex (TC) followed by binding to the 40S ribosomal subunit. Phosphorylation of the α subunit of eIF2 at Ser-51 sequesters it in an inactive GDP-bound complex with its GTP-exchange factor, eIF2B, resulting in attenuation of translation initiation [9]. Phosphorylation of eIF2α can occur in response to the accumulation of unfolded proteins in the ER lumen to limit protein synthesis and relieve the ER load [10]. Still, gene expression is required for translating genes that help cells cope with ER stress and also resume normal cell function. Thus, eIF2α phosphorylation favors the translation of the transcription factor ATF4, which triggers a transcriptional response to cope with ER and oxidative stress. Further, in a negative feedback loop, eIF2α signaling induces GADD34, the regulatory subunit of the serine/threonine protein phosphatase 1 (PP1C), which directs PP1C to dephosphorylate its substrate eIF2α. This allows cells to restore normal eIF2α phosphorylation and normal protein synthesis [11,12].

Cancer cells can enhance general and preferential translation of specific mRNAs coding forproteins that stimulate proliferation or inhibit apoptosis (reviewed in [13]). For example, ErbB2 can enhance c-Myc protein synthesis[14] and increase Src translation via the Akt/mTOR/4EBP-1 pathway [15]. In addition, ErbB2 itself is post-transcriptionally regulated [16-18]. Further, Ras signaling, which ErbB2 can activate, can cause robust recruitment of mRNAs for efficient translation even if transcription of the same mRNAs is not enhanced [19]. Thus, increased translation of multiple mRNAs may allow oncogenes to rapidly stimulate growth and/or adapt to growth/survival conditions. However, how ErbB2 signaling cross talks with eIF2α signaling has not been investigated in depth.

The role of eIF2α in oncogenesis has been established by studies which demonstrated that overexpression of a non-phosphorylatable mutant of eIF2α[20] or a dominant-negative eIF2α kinase [21] were sufficient to transform cells. We showed that persistent phosphorylation of eIF2α by its upstream kinase PERK inhibits proliferation of human mammary epithelial and squamous carcinoma cells exerting growth suppressive effects *in vivo *[22,23]. We also showed that enhancement of eIF2α phosphorylation with the small molecule inhibitor of GADD34-PP1c complex, salubrinal, can inhibit tumor growth of head and neck carcinoma HEp3, colon carcinoma SW620 and multiple myeloma RPMI8226 and U266B1 cells [23,24]. Here we sought to determine whether (i) signaling by ErbB2 might modulate eIF2α phosphorylation and/or (ii) whether enhancement of eIF2α phosphorylation might limit growth by ErbB2 oncogenic signaling. We studied these possibilities in MCF10A cells overexpressing wild type or constitutively activated (CA) ErbB2 and in primary cultures from MMTV-Neu mouse tumors. These oncogenes lead to phenotypes similar to DCIS (WtErbB2-MCF10A and MMTV-Neu) and invasive lesions (MCF10A-CA-ErbB2) where hyper-proliferation and disruption of mammary acinar morphogenesis are observed.

## Results

### ErbB2/Neu disruption of normal mammary acinar morphogenesis

**Figure 1:**
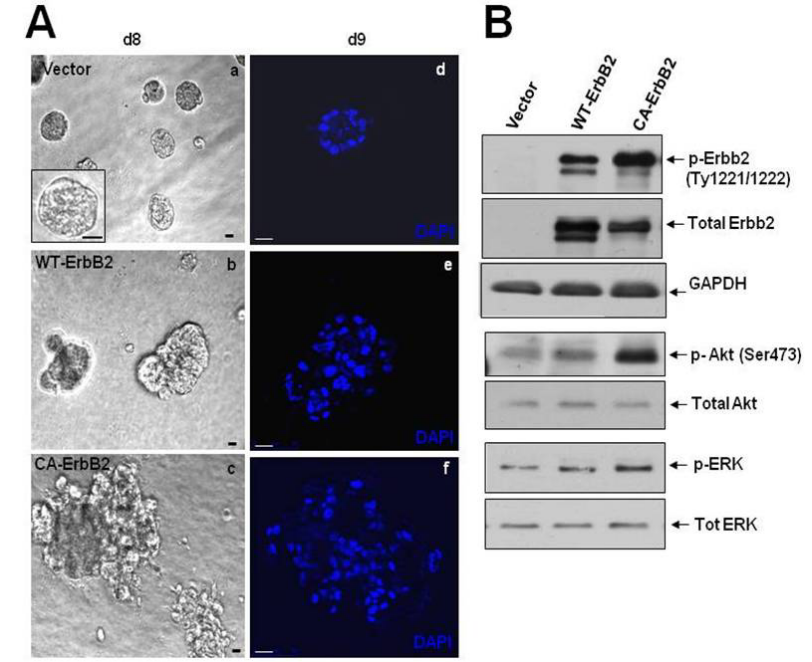
**Characterization of ErbB2 overexpression in normal MCF10A mammary epithelial cells**. **(A) **Phase-contrast micrographs **(a, b, c) **or representative DAPI-stained equatorial confocal sections **(d, e, f) **of acinar structures formed from stable MCF10A cells infected with retrovirus expressing empty vector or the wild-type rat Neu/ErbB2 (Wt-ErbB2) or the constitutively active V664E Neu/ErbB2 mutant (CA-ErbB2) after 8-9 days of growth in Matrigel. Scale bars = 20 μm. **(B) **Western blots of whole-cell lysates from monolayer cultures of MCF10A cells expressing empty vector, Wt-ErbB2 or CA-ErbB2 constructs probed with the indicated antibodies to downstream targets of ErbB2 signaling. GAPDH was used as loading control.

We generated MCF10A cells stably expressing either a wild-type *c-neu *proto-oncogene (the rat homolog of the human *Her2/c-erbB2 *gene, henceforth Wt-ErbB2) or a constitutively active variant of *c-neu *(henceforth CA-ErbB2). The CA-ErbB2 form has a single point mutation in the trans-membrane domain of the receptor, which results in a Val to Glu substitution at position 664 [25] and increased transforming capacity compared to the wild-type receptor [26-28]. Control MCF10A cells (empty vector), formed spheroids with a hollow lumen surrounded by a polarized basal layer of cells in Matrigel (Fig. 1A, **panels a, d**). In contrast, expression of Wt-ErbB2 resulted in much larger, lumen-filled acinar structures by day 9 in Matrigel (Fig. 1A, **panels b, e**). These structures although clearly disorganized and larger were confined and did not show outward invasion into the surrounding matrix. These resembled ductal carcinoma *in situ *(DCIS) structures where there is loss of epithelial organization but no invasion of the surrounding tissue[6,29] (Fig. 1A**panel b**). Expression of CA-ErbB2, resulted in even larger, highly disorganized, lumen-filled multi-acinar structures (Fig. 1A, **panels c, f**), suggesting a dose-dependent effect of ErbB2 signaling on deregulation of acinar development. These structures frequently showed cells invading the surrounding matrix (Fig. 1A**panel c**). Thus, the CA-ErbB2 expressing cells appear to be to some extent more migratory and invasive, like observed in invasive carcinomas [6,29]. Immunoblots confirmed that the Neu/ErbB2 protein was highly expressed in both Wt and CA-ErbB2 cell lines (Fig. 1B). Further, ErbB2 phosphorylation at Y-1221 and Y-1222 residues, known to be auto-phosphorylated during homodimerization of ErbB2 receptors, were readily detected [30]. The levels of total ErbB2 were slightly higher in Wt- than CAErbB2 cells. Still ErbB2 phosphorylation at residues pTy1221/1222 was strong (Fig. 1B). ErbB2 over-expression was functional since we observed an approximately 6-fold increase in P-Ser 473 of Akt, and a 2.4 fold increase in P-Erk 1/2 levels in CA-ErbB2 cells compared to vector control (Fig. 1B), indicating that overexpression of ErbB2/Neu receptors initiated downstream mitogenic signaling. With this model that provides defined pathways readouts and phenotypic endpoints we tested whether eIF2α signaling was modulated by the oncogenes and whether forced enhancement of eIF2α phosphorylation affects ErbB2 modulation of acinar morphogenesis in this system.

### Effect of ErbB2 activation on basal eIF2α phosphorylation and expression of ATF4 and CHOP/GADD153

**Figure 2:**
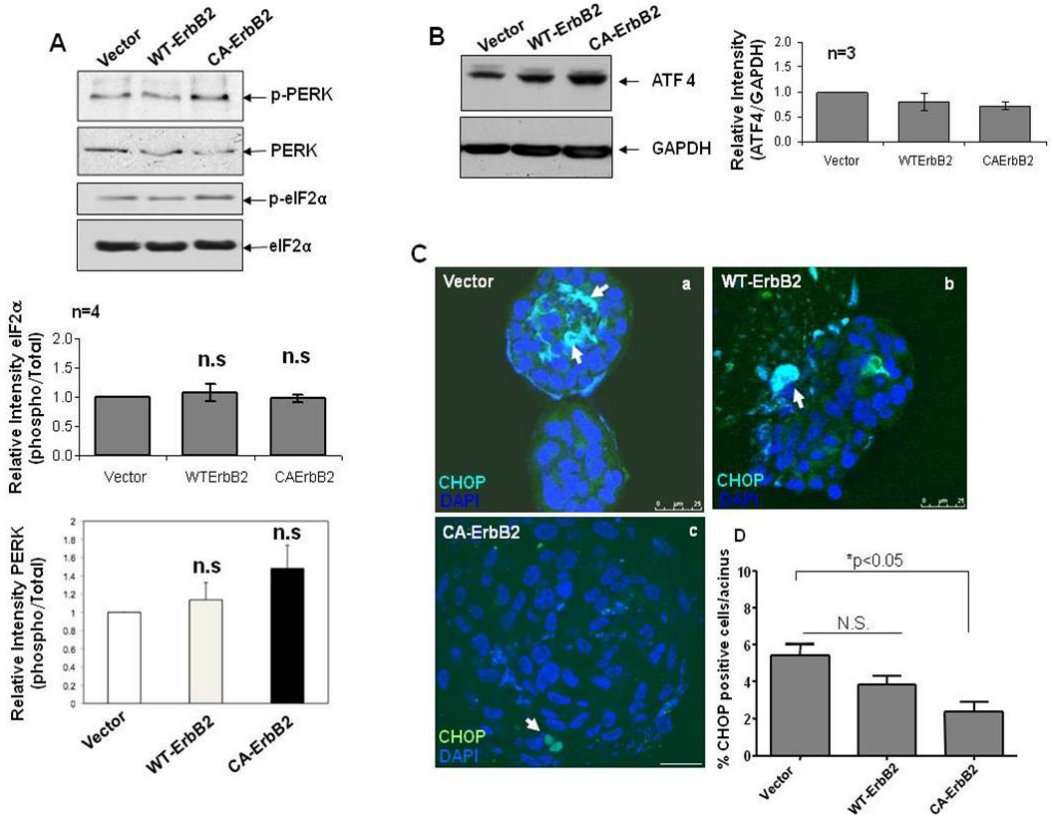
**ErbB2 overexpression does not modulate phospho-eIF2α signaling under basal growth conditions**. **(A, B) **Western blots of whole-cell lysates from monolayer cultures of MCF10A cells expressing empty vector, Wt-ErbB2 or CA-ErbB2 constructs for phospho-and total PERK (A, top panels) or phospho- and total eIF2α (A, bottom panels) or ATF4. Total eIF2α or GAPDH was used as loading control respectively. Bottom panels depict relative intensities quantified by scanning densitometry and normalized to vector control. The averages of three or more independent experiments are shown; ± SEM. **(C) **Equatorial confocal sections of 10-day vector control (**a**) or wild type (Wt) ErbB2/Neu (**b**) or constitutively activated (CA) ErbB2/Neu (**c**) structures labeled with an anti-CHOP antibody (green) or DAPI nuclear stain (blue). Arrows point to CHOP-positive cells in the acinar structure. Scale bars = 25 μm. **(D) **Graph showing the percentage of cells per acinar structure that stained positive for CHOP. Values are representative of four independent experiments performed on different days (8-10) of growth in Matrigel; mean ± SEM. Statistical significance was determined using the unpaired t-test with p < 0.05 defined as statistically significant. N.S - not significant.

Using the model described in Fig. 1 we tested whether eIF2α signaling was modulated by ErbB2 overexpression. We observed no significant modulation of phospho-eIF2α or total eIF2α levels in either Wt or CA-ErbB2 cells compared to control cells as measured by immunoblotting (Fig. 2A). Although we observed a trend towards enhanced PERK phopshorylation in the CA-ErbB2 cells vs. Wt-ErbB2 and β-Gal cells it was not statistically significant when comparing at least 4 independent experiments (Fig. 2A). In agreement, with these results, we found no significant changes in basal ATF4 protein levels between Wt or CA-ErbB2 cells and control cells (Fig. 2B). Thus, ErbB2 signaling in 2D mmonolayers does not seem to significantly modulate the basal phosphorylation of PERK, eIF2α and ATF4 expression for deregulation of acinar morphogenesis.

We next determined whether expression of the downstream target of eIF2α signaling CHOP/GADD153 might be modulated by ErbB2 overexpression in MCF10A cells. Acini that were grown for 8-10 days in Matrigel, when luminal clearing is initiated and apoptosis is evident [31], were stained for CHOP expression. As reported previously [22], we could detect CHOP positive cells within vector control acinar structures and the signal largely localized within the central luminal space. We next used LSCM to quantify the number of CHOP positive cells per acini. A slight but statistically non-significant trend towards less CHOP staining was observed in non-invasive WtErbB2 structures compared to control acini and this signal was randomly localized in the acini, possibly due to the already deregulated architecture of these structures (Fig. 2D). In contrast, invasive CA-ErbB2 cell aggregates showed a statistically significant reduction in the number of cells positively stained for CHOP (Fig. 2D). We conclude that increased ErbB2 signaling while not affecting basal phosphorylation of PERK and eIF2α or ATF4 expression, appears to downregulate CHOP expression in the more invasive structures induced by CA-ErbB2.

### Salubrinal increases phospho-eIF2α levels and significantly restricts ErbB2-deregulated acinar growth

**Figure 3:**
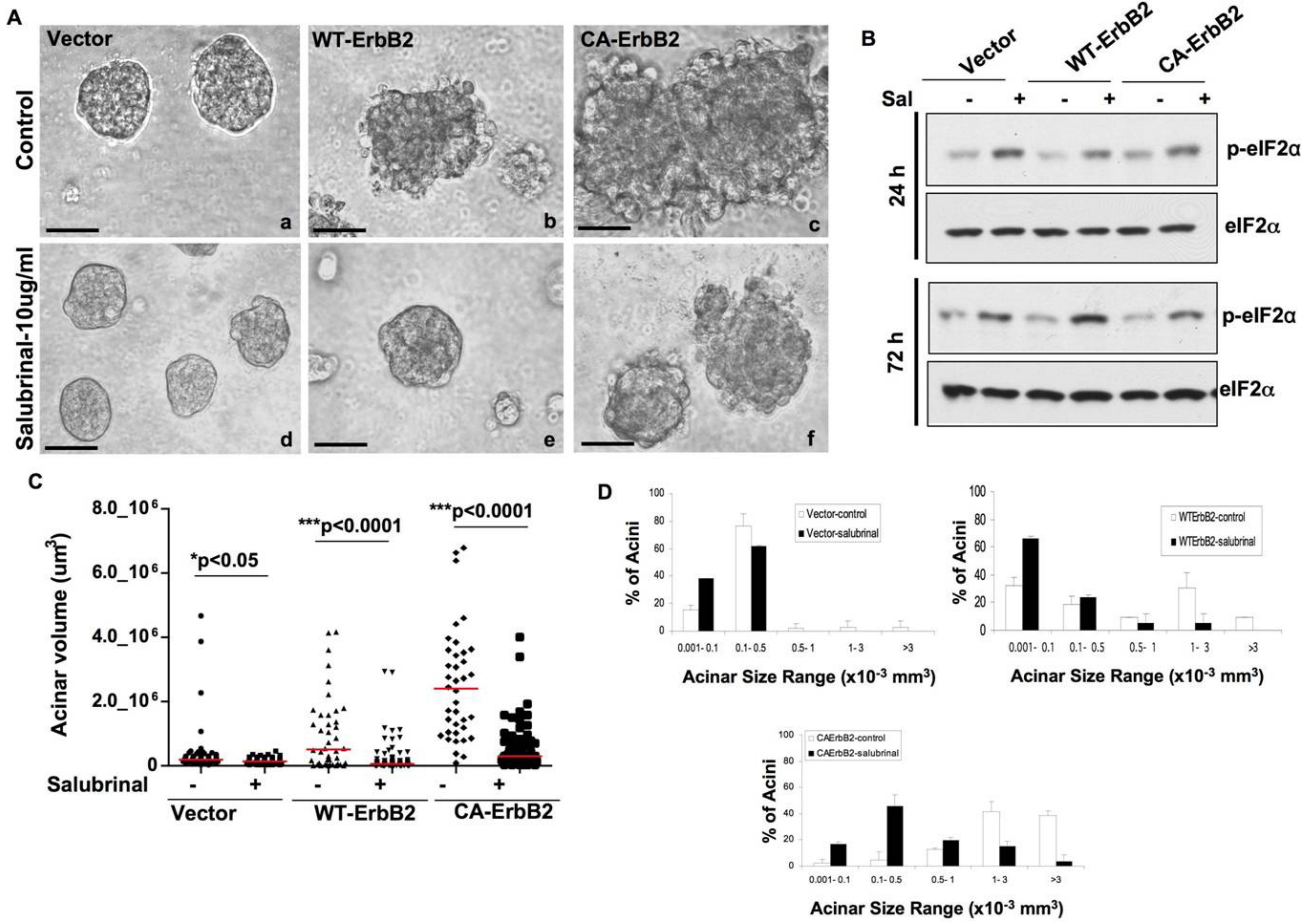
**Salubrinal increases basal phospho-eIF2α levels and significantly limits ErbB2-induced acinar growth**. **(A) **Phase-contrast micrographs of control and ErbB2-expressing acini untreated (top panels) or treated with salubrinal (10 μg/ml) from Day 4-day 10 in Matrigel (bottom panels). Note the reduced acinar size of salubrinal treated ErbB2-expressing acini. Scale bars = 60 μm. **(B) **Western blots showing the increase in phospho-eIF2α levels in empty vector, Wt-ErbB2 or CA-ErbB2 cells untreated or treated with salubrinal under adhered monolayer conditions for the indicated time points. Total eIF2α was used as loading control. **(C) **Acinar volume was quantified on day 8 in Matrigel after daily treatment with salubrinal (day 4 onwards) for vector, Wt-ErbB2 or CA-ErbB2 acini. Acinar volume was estimated by measure two perpendicular diameters per acinus, assuming an ellipsoid structure. Between 50-100 acini were quantified and results are representative of experiments repeated at least three times. Statistical significance was determined using the unpaired t-test with p < 0.05 defined as statistically significant. **(D) **Graphs showing the distribution of acinar sizes over several volume ranges to depict the increase in acinar size upon ErbB2 activation, mean ± SD. Note that salubrinal treatment causes a shift in the percentage of acini falling within the smaller size ranges.

The downregulation of CHOP in CA-ErbB2 structures suggested that perhaps ErbB2 signaling uncouples eIF2α phosphorylation from CHOP induction and its growth inhibitory function. However, the cells might still be sensitive to higher levels of eIF2α phosphorylation. Thus, we next tested whether ErbB2 oncogenic signaling could be counteracted upon forced eIF2α phosphorylation or whether it might still bypass the growth inhibitory function of this pathway [23,32,33]. To this end we used Salubrinal, a recently discovered small molecule inhibitor shown to disrupt the GADD34-PP1 phosphatase complex [34] and persistently induce high P-eIF2α levels. Four days after seeding in Matrigel cells were treated with a low dose of salubrinal (10 μg/ml) until days 8 or 10. Treatment with salubrinal at 24 and 48 hrs did not compromise cell viability (see additional File 1B) but negatively affected growth of Wt and CA-ErbB2 acini in adhered monolayer conditions (see additional File 1C). This effect was much more striking in Matrigel, where we observed that salubrinal treatment significantly reduced acinar size and growth in Matrigel (Fig. 3A). Compared to untreated controls, salubrinal-treated Wt-ErbB2 cells formed smaller, more organized growth-arrested acini and also appeared to restore to some extent their normal organization. In CA-ErbB2 structures salubrinal treatment significantly reduced overall acinar size but did not seem to almost fully restore normal acinar architecture and block local invasion. Salubrinal treatment did not grossly affect the growth and organization of vector control acini, although a slight reduction in overall size was observed (Fig. 3A, D). We confirmed that treatment with this low dose of salubrinal (10 μg/ml) was sufficient to enhance basal eIF2α phosphorylation (over 2.5-fold) in vector, Wt and CA-ErbB2 cells, as early as 24 hrs after treatment and which could be maintained with daily salubrinal treatment up to 72 hrs (Fig. 3B). Attempts to detect enhanced phopshorylation of eIF2α in 3D acini showed a general staining pattern throughout the acini which was not specific for basal or luminal cells and enhancement through tunicamycin showed an enhanced signal that could not be specifically assigned to luminal or basal cells, making the results based on staining intensity more difficult to interpret (data not shown). To estimate the size differences between 8-day old vector, Wt-ErbB2 and CA-ErbB2 acini, two perpendicular acinar diameters were measured using calibrated software [22] and the volumes of individual acini were calculated, considering an ellipsoid morphology (Fig. 3C). The large acinar size in Wt-ErbB2 and CA-ErbB2 (Fig. 3C) was markedly reduced by persistent treatment with salubrinal. Quantification of the distribution of acinar sizes over several volume ranges showed that in untreated vector controls, less than 5 percent of acini exceeded the 0.5-1 × 10^-3 ^mm^3 ^volume range (Fig. 3D) and salubrinal treatment maintained acinar size below this range (Fig. 3D). In contrast, a large proportion (between 10-40 percent) of untreated Wt and CA-ErbB2 acini exceeded the 0.5-1 × 10^-3 ^mm^3 ^volume range. Treatment with salubrinal considerably reduced the frequency of these larger acini and shifted the majority of acini towards the smaller volume ranges (less than 0.5 × 10^-3 ^mm^3^) (Fig. 3D). These findings demonstrate that ErbB2-induced deregulation of mammary acinar development can be suppressed by inhibiting eIF2α dephosphorylation with salubrinal.

### Differential proliferative and apoptotic responses to Salubrinal by Wt vs. CA-ErbB2 expressing acini

**Figure 4:**
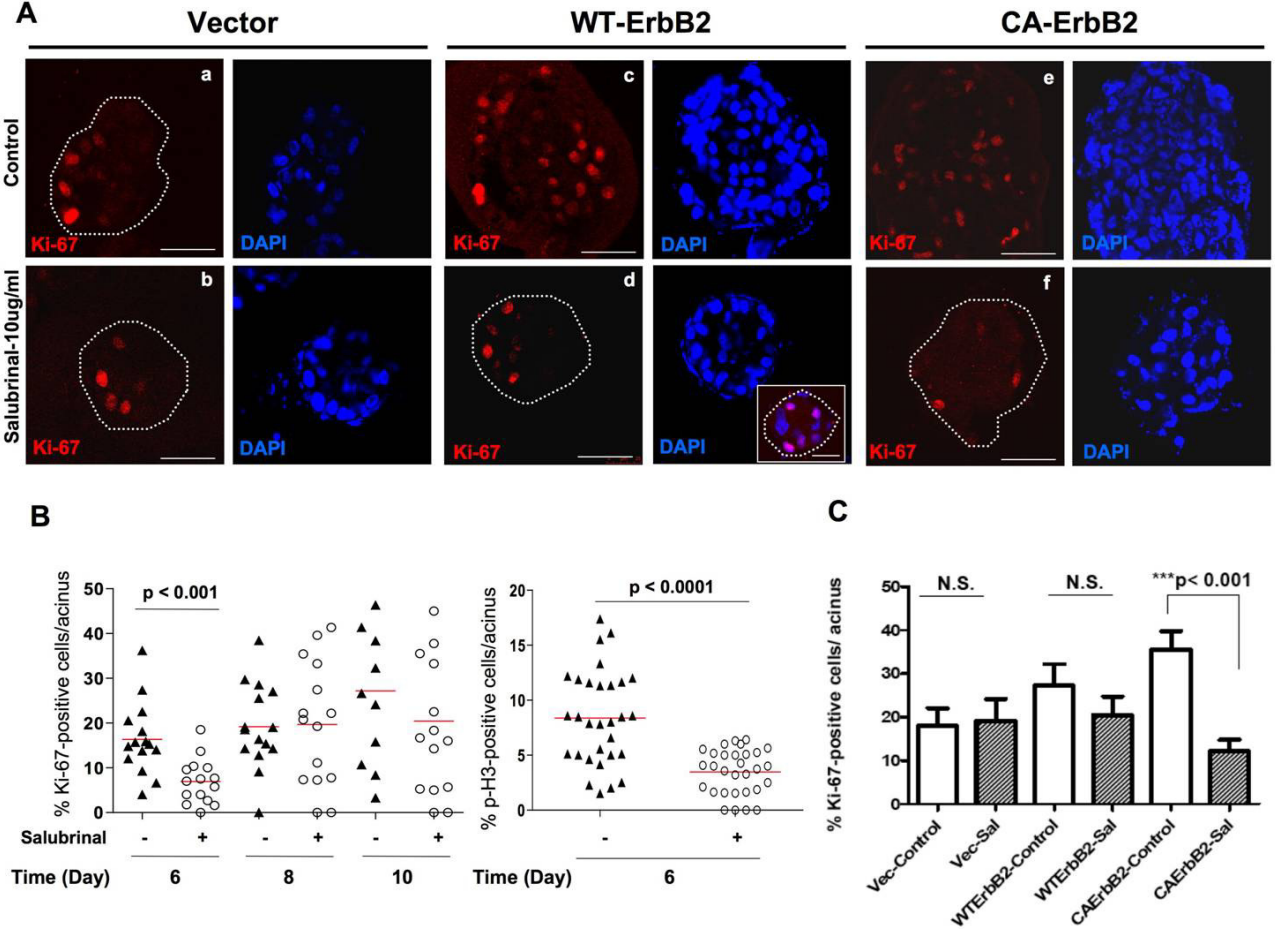
**Induction of eIF2α phosphorylation by salubrinal inhibits ErbB2-induced hyperproliferation**. **(A) **Representative confocal images of equatorial cross-sections through day 10 vector control, wild type and constitutively active ErbB2 acini stained for Ki-67 (red) or the corresponding images stained with DAPI (blue). Untreated acini (**panels a, c and e**) and salubrinal treated acini (**panels b, d and f**). Note Ki-67-positive cells in the outer basal layer of cells in untreated vector control acini (outlined in dotted lines in some structures where edges are not obvious) compared to those occupying the central luminal space in untreated ErbB2-over-expressing acini. Scale bars = 40 μm. Inset (**panel d**) depicts a smaller salubrinal-treated Wt-ErbB2 acinus with many Ki-67 positive cells. **(B) **Graph showing distribution of the percentage of wt-ErbB2 cells per acinus that stained positively for Ki-67 at days 6, 8 and 10 (left panel) or phospho-H3 at day 6 (right panel); horizontal red line indicates the median. Statistical significance was determined using the unpaired t-test with p < 0.05 defined as statistically significant. N.S - not significant. Salubrinal treatment significantly decreases the percentage of Ki-67 positive cells in WT-ErbB2 acini only early at day 6.**(C) **Graph showing distribution of the percentage of cells per acinus that stained positively for Ki-67 cells; mean ± SEM. Statistical significance was determined using the unpaired t-test with p < 0.05 defined as statistically significant. N.S - not significant. Note the increase in percentage of Ki-67 positive cells in untreated CA-ErbB2 expressing acini compared to vector controls. Salubrinal treatment significantly decreases the percentage of Ki-67 positive cells in CA-ErbB2 acini.

The decrease in acinar sizes observed with salubrinal could result from decreased cell proliferation, increased apoptosis or a combination of both. To test whether salubrinal caused a reduction in cell proliferation, vector, Wt and CA-ErbB2 acini were treated with salubrinal from day 4-10 in Matrigel, fixed and stained for Ki-67 (proliferation marker). The percentage of Ki-67 positive cells per acinus was estimated using standard immunofluorescence and LSCM [22]. Untreated vector acini showed Ki-67 positive cells mainly located in the basal layer of epithelial cells (Fig. 4A, **panel a**) and this was not affected by Salubrinal treatment (Fig. 4A, **panel b**) suggesting that the slight decrease in vector acini size is not attributable to reduced proliferation. CA-ErbB2 overexpression caused a noticeable increase in the percent of Ki67 positive cells compared to empty vector control acini (Fig. 4A, **panels c, e and **4B). In all cases Ki67 positive cells were found both in the outer rim as well as among the cells occupying the central luminal space (Fig. 4A, **panel c, e**). Salubrinal treatment of Wt-ErbB2 acini caused a statistically significant reduction in Ki67 (S-phase marker) and phospho-H3 (G2/M marker) staining at day 6 of acinar growth, suggesting an initial inhibition of proliferation (Fig. 4B, **right and left panels**). However, these differences disappeared at day 8 and day 10 (Fig. 4B-C), suggesting that at these time points salubrinal only marginally impacts proliferation; we could also detect smaller sized acini still containing many positively stained cells (Fig. 4A-d**inset, DAPI channel and **4C). In contrast, a statistically significant decrease in Ki-67 staining was observed in CA-ErbB2 acini (Fig. 4C). Thus, it appears that both wt-ErbB2 and CA-ErbB2 are susceptible to salubrinal inhibition of proliferation, except that this effect is only observed during early phases of acinar development in wt-ErbB2 cells and throughout the growth phases for CA-ErbB2 cells.

**Figure 5:**
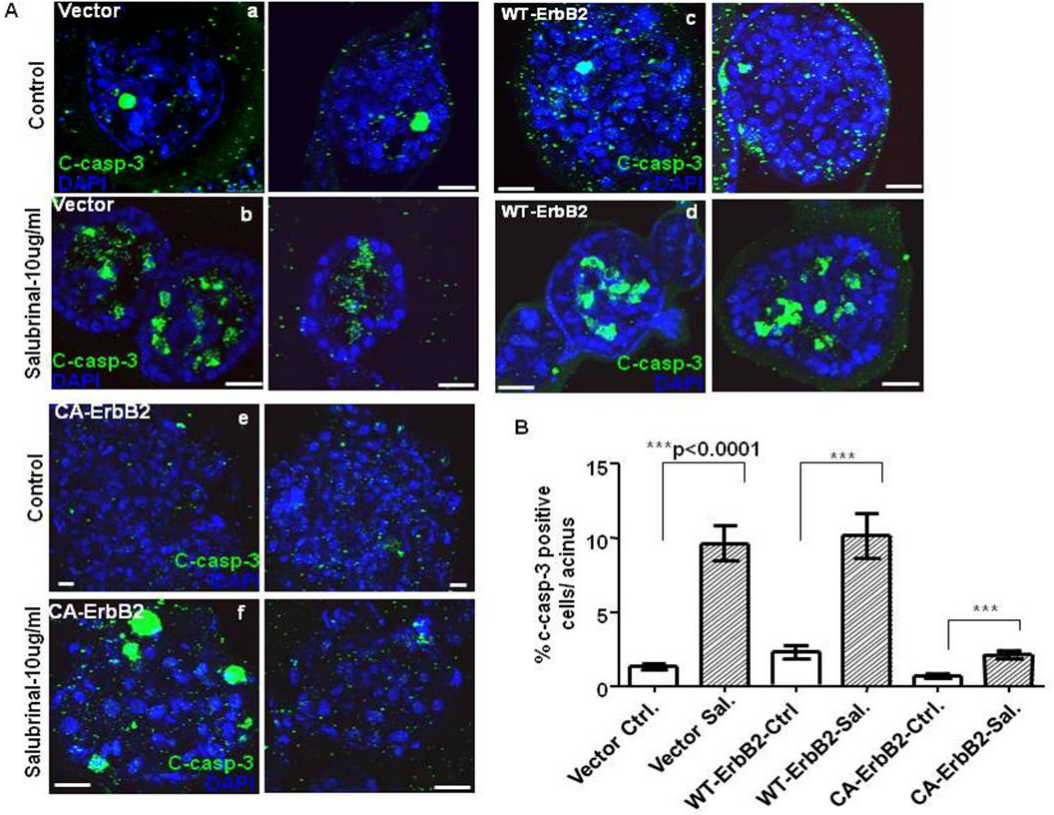
**Salubrinal treatment induces apoptosis and accelerates luminal clearing in control and wild type ErbB2 acini**. **(A) **Two representative confocal images of equatorial cross-sections of day 10 vector control, wild type and constitutively active ErbB2 acini stained for cleaved caspase-3 (c-casp-3; green) or DAPI (blue). Untreated acini (**panels a, c and e**) and salubrinal treated acini (**panels b, d and f**). Note increase in c-caspase-3 stained cells within the luminal space in salubrinal-treated vector and Wt-ErbB2 acini. Scale bars = 25 μm. **(B) **Graph showing distribution of the percentage of cells per acinus that stained positively for c-caspase3; mean ± SEM. Statistical significance was determined using the unpaired t-test with p < 0.05 defined as statistically significant. N.S - not significant.

In Wt-ErbB2 acini decreased acinar size could only be partially explained by reduced proliferation in response to salubrinal treatment. Thus, we tested whether apoptosis was being modulated by sustained eIF2α phosphorylation. In this case cleaved caspase-3 staining was used as a marker of apoptosis [22]. Our results revealed that sustained treatment with salubrinal resulted in a significant increase in cleaved caspase-3 positive cells that localized mainly to the central luminal space in vector control and Wt-ErbB2 acini (Fig. 5A, **panels b, d**) and at a lower frequency along the periphery in CA-ErbB2 acini (Fig. 5A, **panel f**). Quantification of the percentage of cleaved caspase-3 positive cells per acinus revealed that vector and Wt-ErbB2 acini displayed similar rates of apoptosis under basal untreated conditions while constitutively active ErbB2 signaling displayed approximately 1.5-fold less cell death further highlighting the stronger potency of this mutant ErbB2 receptor (Fig. 5B). Salubrinal treatment significantly enhanced the number of cleaved caspase-3 positive cells/acini in all three-cell types, albeit to different extents. In vector and in Wt-ErbB2 acini the induction was between 5-8 fold. In contrast, in salubrinal-treated CA-ErbB2 acini, the fold induction of apoptosis was much lower (approximately 3-fold) compared to untreated cells, although still statistically significant (Fig. 5B). Of note is the fact that although salubrinal enhanced luminal apoptosis in Wt-ErbB2 structures, this did not seem to affect the basal layer of viable cells but rather accelerated luminal apoptosis, a normal occurrence during acinar development. Thus, these structures that resemble DCIS can be forced into a more differentiated or normal architecture by enhancing eIF2α phosphorylation Salubrinal did not affect the levels of BimEL, a pro-apoptotic protein implicated in lumen formation [7] (data not shown) indicating that apoptosis might proceed via selective activation of other pro-apoptotic phospho-eIF2α targets or via autophagic death [35]. Our results suggest that salubrinal treatment can counteract the ErbB2-mediated deregulation of morphogenesis in 3D-culture by inhibiting early proliferation, accelerating apoptosis and enhancing luminal clearing in Wt-ErbB2 over-expressing cells. In CA-ErbB2 cells, inhibition of proliferation is primarily responsible for reduced acinar growth and size.

**Figure 6:**
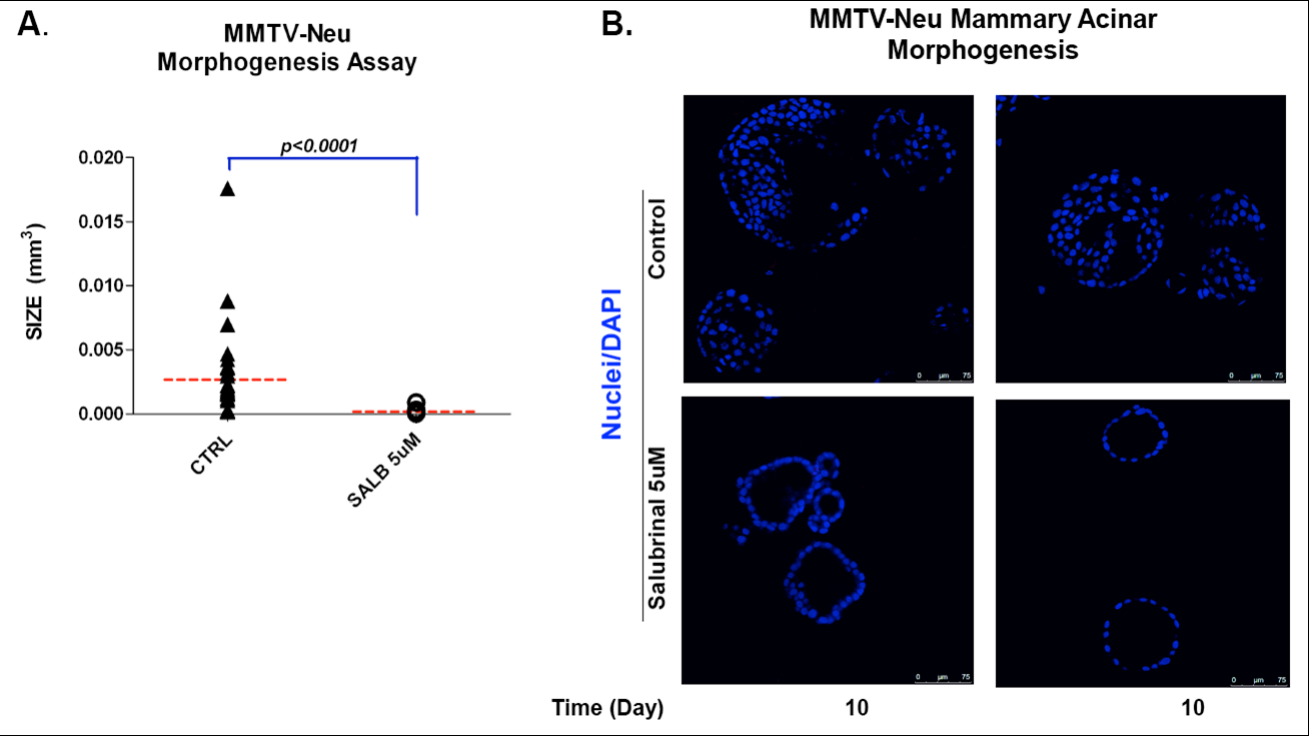
**Salubrinal normalizes the deregulated acinar morphogenesis of MMTV-Neu mammary tumor cells**. Single epithelial cells isolated from MMTV-Neu mouse mammary tumors were grown in 3D organotypic/Matrigel cell culture over a period of 10 days. **(A) **Size of vehicle control (CTRL) and salubrinal 5 ug/ml treated acini was calculated following the equation [(Length × width^2^)/2 = acini volume (mm^3^)] and plotted; Mann Whitney test p < 0.0001, n = 30. **(B) **Representative confocal images of equatorial sections through control and salubrinal treated MMTV-Neu acini stained with DAPI at day 10 in Matrigel. Salubrinal treatment reverted the tumorigenic phenotype of MMTV-Neu acini by promoting formation of normal lumens in growth arrested acini (lower panel) as compared to untreated acini showing luminal filling and increased size (upper panels).

We next tested whether the effect of salubrinal was limited to the MCF10A model. Treatment with salubrinal of control normal mammary epithelial cells from FVB mice were grown in Matrigel. In these cells, like in parental MCF10A, salubrinal had minor effects on morphogenesis in 3D (not shown). Further, in agreement with our results in MCF10A cells overexpressing ErbB2, MMTV-Neu cells obtained from spontaneous tumors that arose in these mice formed dramatically enlarged structures devoid of proper tissue architecture and with a filled lumen (Fig. 6). However, treatment with salubrinal strongly inhibited expansion of these deregulated structures reducing their size in a statistically significant manner and favoring cavitation and proper lumen formation (Fig. 6A).

### Salubrinal-mediated inhibition of acinar growth correlates with increased expression of CHOP and reduced cyclin D1 levels

**Figure 7:**
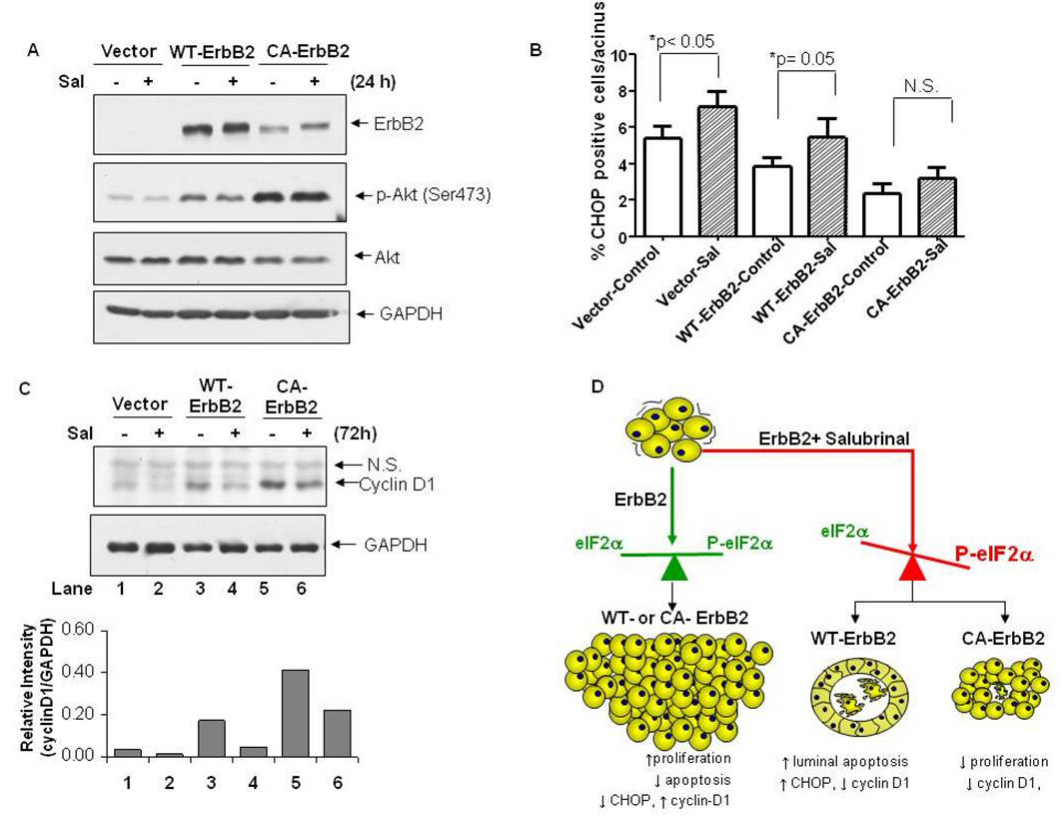
**Salubrinal may restrict growth by increasing expression of CHOP and suppressing cyclin D1 levels**. **(A) **Western blots for total ErbB2, phospho-Ser473 and total Akt levels after 24 hr salubrinal treatment (10 μg/ml) of cells grown in monolayer conditions. GAPDH was used as loading control. **(B) G**raph showing quantification of the percentage of CHOP positive cells per acinus, mean ± SEM. Statistical significance was determined using the unpaired t-test with p < 0.05 defined as statistically significant. N.S - not significant. Note the modest increase in CHOP expression in salubrinal-treated vector control and Wt-ErbB2 acini but not in CA-ErbB2 acini. **(C) **Western blots (top panels) for cyclin D1 levels after 72 hr salubrinal treatment (10 μg/ml)of cells grown in monolayer conditions. GAPDH was used as loading control, N.S-non-specific band. Bottom graph, Cyclin D1 protein levels were quantified by scanning densitometry and normalized to GAPDH.**(D) **Schematic depicting the growth-inhibitory effects of increased eIF2α phosphorylation on aberrant acinar morphogenesis induced by ErbB2.

We first tested whether salubrinal might affect total ErbB2 protein levels. Western blot analysis showed that salubrinal had no effect on ErbB2 protein expression or its downstream targets Akt and Erk1/2 (Fig. 7A and data not shown) indicating that the growth inhibitory effect of salubrinal is not due to a decrease in total ErbB2 or downstream target protein levels. Salubrinal did not modulate phospho-Ser473-Akt (Fig. 7C) or phospho-T202/Y204-Erk1/2 levels at 24, 72 or 96 hrs (data not shown), indicating that its growth suppressive effects were not due to reduced activation or non-specific regulation of these signaling components.

CHOP induction is dependent of eIF2α phosphorylation, mediates ER stress-induced growth arrest and apoptosis and can be up-regulated by salubrinal treatment [34,36]. CHOP staining was used as surrogate phenotypic readout for phospho-eIF2α signaling and the number of CHOP positive cells per acinus was quantified using LSCM as in Fig. 2. Our results showed a statistically significant increase in the frequency of CHOP-positive cells in vector and Wt-ErbB2 acini after salubrinal treatment (Fig. 7B). In contrast, CA-ErbB2 acini were able to bypass the salubrinal-mediated induction of CHOP. These results indicate that the increase in CHOP expression coincides with the induction of acinar apoptosis in vector acini and Wt-ErbB2 structures. In CA-ErbB2-expressing cells this mutant oncogene appears to block apoptosis and this associates with less CHOP induction. This suggests that prolonged treatment with salubrinal might induce a state of quiescence in these cells.

Cyclin D1 has previously been shown to be a major mediator of ErbB2-induced tumor cell proliferation and oncogenesis [37]. When compared to vector control MCF10A cells, cyclin D1 levels were significantly increased in Wt and to an even greater extent in CA-ErbB2 cells (Fig. 7C). This paralleled the increased rates of proliferation in 3D cultures (Fig. 4B). Cyclin D1 levels were previously shown to be downregulated upon treatment with a derivative of salubrinal (sal003) and by inducible activation of a dimerizing Fv2E-PERK protein [34,36]. We thus determined whether reduced cell proliferation was partly attributable to a salubrinal-induced decrease in cyclin D1 levels. We found that sustained salubrinal treatment for 72 hrs resulted in a 3.6-fold and approximately 1.9-fold decrease in cyclin D1 protein levels in treated Wt-ErbB2 and CA-ErbB2 cells, respectively, compared to untreated cells (Fig. 7C). This decrease in cyclin D1 was also evident as early as 24 hrs post treatment (data not shown). We conclude that sustained eIF2α phosphorylation results in the induction of CHOP and downregulation of cyclin D1 which could further account for the reduced proliferation and smaller acinar size in salubrinal-treated ErbB2 acini.

## Discussion

We established a model where acquisition of phenotypes that resembled DCIS or invasive carcinoma can be mimicked by expressing the Wt-ErbB2 and a CA-ErbB2, respectively. In this model we set out to determine whether ErbB2 over-expression was linked to modulation of eIF2α phosphorylation and downstream pathway induction. Our results show that there is a dependence of ErbB2 signaling on eIF2α phosphorylation to promote deregulation of mammary acinar morphogenesis in 3D cultures. We found that both WT- and CA-ErbB2 did not significantly modulate basal PERK or eIF2α phosphorylation. Consistently, the expression of an eIF2α downstream target ATF4 also remained unchanged. Conversly expression of the ATF4 target gene CHOP, was decreased but only in invasive CA-ErbB2 expressing structures. Possible explanations for this uncoupling are that ATF4 activity may be reduced by ErbB2 signaling, that other transcription factors that regulate CHOP expression, such as ATF6 [38] might be inhibited by CA-ErbB2 or that this oncogene induces the rapid degradation of CHOP protein. That CHOP is down regulated by ErbB2 is similar to that observed with Ras and consistent with its function in inducing growth arrest and/or apoptosis [36,39]. Thus, ErbB2 signaling may uncouple eIF2α-ATF4 signaling from the deleterious effects of CHOP.

We found that as opposed to normal MCF10A or mouse MECs, ErbB2 overexpressing human or mouse (MMTV-Neu) MECs were very sensitive to enhanced eIF2α phosphorylation. This was supported by the fact that Salubrinal treatment and increased eIF2α phosphorylation strongly inhibited Wt- or CA-ErbB2 deregulation of MCF10A acinar morphogenesis. This was also evident in MMTV-Neu induced structures that cavitated and reduced their size upon Salubrinal treatment. However, depending on the intensity of ErbB2 signaling, salubrinal treatment achieved inhibition of the deregulated acinar morphogenesis via different mechanisms. In Wt-ErbB2 over expressing acini, which resemble structures observed in DCIS, salubrinal treatment was largely associated with early inhibition of proliferation (days 4-6) that was lost at later time points (days 8-10) of morphogenesis when a strong induction of apoptosis was detected. Interestingly, this apoptosis occurred mostly in the luminal population of cells, stressing the specificity of this signal to the luminal compartment. Notably, salubrinal treatment could accelerate the normal process of luminal clearing which fails to occur during ErbB2 oncogenesis [7]. These results also suggested that salubrinal did not randomly exert toxic effects on all cells within the acini but that it rather accelerated luminal clearing without compromising the viability of basal cells within these structures. The induction of apoptosis also correlated with enhanced luminal cleaved caspase-3 positive cells and enhanced CHOP induction. Further, studies are required to elucidate the precise apoptotic pathways that are activated by salubrinal treatment. Together these findings suggest that ErbB2 positive cancer cells in lesions like DCIS might be particularly sensitive to therapeutic drugs that enhance eIF2α phosphorylation.

In CA-ErbB2 acinar structures, which resembled invasive lesions, salubrinal treatment also strongly inhibited growth but mainly through inhibition of proliferation. Apoptosis although induced, was of a much lower magnitude. Activated ErbB2 cells showed stronger activation of Akt signaling that was not affected by salubrinal treatment (Figs. 1 and 6). This suggests that enhanced Akt signaling might in part allow these cells to resist the growth-inhibitory effects of salubrinal treatment possibly by entering a state of quiescence. The decrease in cyclin D1 levels in CA-ErbB2 cells was also of a lower magnitude compared to Wt-ErbB2, suggesting that the growth arrest was not entirely dependent on down-regulation of this cell cycle protein. Total protein levels of phospho- or total ErbB2, Akt and Erk1/2 were unchanged, highlighting the selective and specific mode of action of salubrinal. However, we can not rule out that other target genes for GADD34-PP1C [40] might be affected by salubrinal. It will be important to determine whether phospho-mimetic mutants of eIF2α or the use of the FV2E-PERK fusion protein also generate the same effects as salubrinal.

We recently showed that chronic inhibition of eIF2α phosphorylation by PERK dominant negative mutants in MCF10A cells results in acinar deregulation and hyperplastic benign growth *in vivo *[22]. Thus, perturbations that ablate the growth inhibitory function of PERK-eIF2α signaling can favor benign tumor formation. Here we show that preventing eIF2α dephosphorylation can severely compromise the oncogenic effects of ErbB2 signaling. Together these studies support the notion that increases in eIF2α phosphorylation and signaling above a certain threshold can be growth inhibitory in cancer cells. Our data allows us to entertain the possibility that the process of transformation requires cells to dynamically remodel eIF2α phosphorylation status to favor survival and cells compromised in the ability to do so (i.e. very strong eIF2α phosphorylation) may be negatively selected. For example, oncogene-induced senescence in normal melanocytes requires eIF2α signaling [41]. It is possible then that only those cells able to adapt the eIF2α pathway to their advantage, that is maintain a more plastic remodeling of the eIF2α pathway without reaching very high or persistent phospho-eIF2α levels, go on to be transformed. We therefore predict that cells that escape ErbB2-induced senescence, might co-opt eIF2α phosphorylation and downstream signaling for survival.

## Conclusion

We conclude that while ErbB2 signaling does not seem to significantly modulate eIF2α phosphorylation at Ser51 tumor cells overexpressing this oncogene are highly sensitive to the effects of hyper-phosphorylated eIF2α levels. In contrast, normal MECs are marginally sensitive to Salubrinal treatment. We recently showed that doses of salubrinal that have no effect on basal multiple myeloma viability greatly sensitize these cells to bortezomib (Velcade) treatment in conjunction or after bortezomib treatment [24]. Our present work describes a previously unrecognized approach for targeting the hyper-proliferation induced by ErbB2 via pharmacological activation of eIF2α signaling. Therapeutic treatments targeting the eIF2α pathway may prove, alone or in combination with other targeted therapies, to be useful in the treatment of cancers over-expressing ErbB2. This and the fact that salubrinal or more powerful derivates like Sal003 do not appear to have significant toxicities in murine models may warrant further investigation into whether Salubrinal or similar molecules that target GADD34-PP1C could be used to treat breast cancers with amplified ErbB2.

## Methods

### Cell culture and generation of stable cell lines

MCF10A cells and the culture conditions in 2D or 3D cultures were described previously [22]. The retroviral vectors pLXSN empty vector, pLXSN-NNeu (Wt rat Neu) and pLXSN-Neu* (constitutively active rat Neu, with a 664 Val->Glu substitution) were obtained from Dr. Lisa Petti (Albany Medical Center, Albany, NY) [25]. Retroviral delivery of transgenes was done as described previously [22]. Primary cultures of control normal mammary epithelial cells from FVB mice or cells from MMTV-Neu transgenic mice (FVB) were prepared as described [42]. For Salubrinal treatment in Matrigel, acini were treated with 10 μg/ml of salubrinal dissolved in DMSO or equal volume of solvent control starting on Day 4 of growth in Matrigel and added fresh daily until Day 10 of growth.

### Reagents and Antibodies

Salubrinal was obtained from Calbiochem. Anti-phospho-ErbB2 (pTy1221/1222), anti-phospho- and total PERK (P-Thr 980) and eIF2α (P-Ser 51), anti-phospho-Akt (Ser473), cyclin D1 and anti-cleaved caspase-3 were from Cell Signaling (Danvers, MA). Anti-total ErbB2 and anti-total Erk 1 was from BD Biosciences. Anti-ATF4, anti-CHOP/GADD153 (sc-575), anti-GADD34 and anti-phospho-Erk 1/2 were from Santa Cruz Biotechnology (Santa Cruz, CA). Anti-Ki-67 was from Invitrogen (Carlsbad, CA) and anti-GAPDH from Calbiochem. Secondary antibodies were described previously[22].

### Western analysis and viability assays

Cells were washed with ice-cold PBS, lysed in RIPA buffer containing protease and phosphatase inhibitors (Roche) and processed for immunoblot analysis as described previously [22]. Protein concentration was determined using the Bradford reagent (Biorad). Cell viability was determined using a Trypan blue exclusion assay.

### Immunofluorescence and Laser Scannin Confocal Microscopy (LSCM)

Processing of MCF10A cells grown in 3D Matrigel for 8-10 days was done as described previously [22]. For detection of CHOP, the primary antibody was used at a concentration of 1:50 (Santacruz Biotechnology, sc-575). Confocal images representing equatorial sections of acinar structures were acquired using standardized acquisition parameters on a Leica TCS SP5 inverted confocal microscope (Leica Microsystems, USA) with 40× or 63× objectives. Phase contrast images of acini were captured using a calibrated Nikon Eclipse TS100 microscope fitted with a digital SPOT-RT camera and corresponding software (Sterling Heights, MI). Acinar size was estimated as described previously [22].

### Statistical analyses

Statistics were performed using MS-Excel or GraphPad Prism 5.0 software (San Deigo, CA) and P values were calculated using one-way ANOVA followed by the Bonferroni multiple comparison post test or the unpaired t test with p < 0.05 considered statistically significant. N.S = not significant.

## Abbreviations

eIF2: eukaryotic translation initiation factor-2; GADD: growth arrest and DNA damage-inducible protein; ER: endoplasmic reticulum; CHOP: C/EBP homologous protein.

## Authors' contributions

SJS: developed transgenic cells, performed a majority of the experiments, interpreted the data and wrote the paper. WHC: performed experiments related to MMTV-Neu tumor and mouse MECs and to detection of Ki67 in WT-ErbB2 cells. AAV: performed experiments related to phospho- and total-PERK detection as well as detection of Ki67 and phospho-H3 in WT-ErbB2 cells. EFF: provided the MMTV-Neu transgenic model and participated in the experimental design and data interpretation of the experiments. JAAG: participated in conceptual and experimental design, interpretation of the data and writing of the paper. All authors read and approved the final manuscript.

## Supplementary Material

Additional file 1 **Supplementary figure 1**. **(A) **Western blot for GADD34 from lysates of cells grown in monolayer conditions. GAPDH was used as loading control. **(B) **Graph showing viability of cells treated with vehicle or 10 μg/ml salubrinal in adhered conditions for the indicated time points, using Trypan blue exclusion. Points indicate average ± SD. **(C) **Graph showing the effect of salubrinal on 2D cellular proliferation. Cells were treated with vehicle or 10 μg/ml salubrinal in adhered conditions for the indicated time points before detachment and counting using a hemocytometer. Bars indicate average ± SD. Click here for file
