# Supplementary figures and images for: Inhibition of eIF2α dephosphorylation inhibits ErbB2-induced deregulation of mammary acinar morphogenesis
Source: BMC Cell Biol. 2009 Sep 15;10:64. doi: 10.1186/1471-2121-10-64 (PMC2754445; doi:10.1186/1471-2121-10-64)

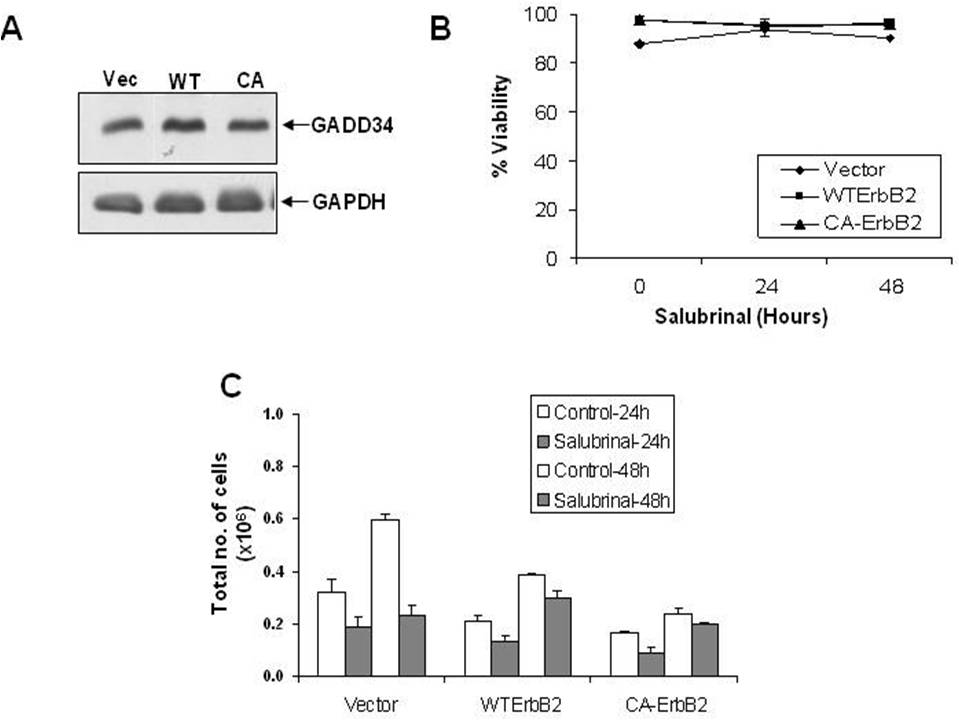

Supplement: Additional file 1 — Supplementary figure 1. (A) Western blot for GADD34 from lysates of cells grown in monolayer conditions. GAPDH was used as loading control. (B) Graph showing viability of cells treated with vehicle or 10 μg/ml salubrinal in adhered conditions for the indicated time points, using Trypan blue exclusion. Points indicate average ± SD. (C) Graph showing the effect of salubrinal on 2D cellular proliferation. Cells were treated with vehicle or 10 μg/ml salubrinal in adhered conditions for the indicated time points before detachment and counting using a hemocytometer. Bars indicate average ± SD. [file 1471-2121-10-64-S1.jpeg]
